# Isn't it time to start speaking about "European surgeons"?

**DOI:** 10.1186/1749-7922-4-27

**Published:** 2009-07-25

**Authors:** Federico Coccolini, Daniel Lazzareschi

**Affiliations:** 1 General, Emergency and Transplant Unit, Sant'Orsola-Malpighi University Hospital, Bologna, Italy; 2 University of Berkeley, Berkeley, CA, USA

## Abstract

**Background:**

Emergency surgery has become a neglected specialization in Europe and in many other parts of the world. In certain medical fields, emergency surgery isn't even considered an autonomous specialization. However every emergency surgeon must have a good formation in General Surgery but exist huge disparities between different European surgical formative systems.

**Methods:**

An analysis of the main problems of the European surgical formative system was conducted.

**Results:**

This discrepancy between formative systems is absolutely unacceptable and presents a notable hazard for the European Union, considering that surgical certifications are reciprocally recognized between programs within all European Union states.

**Conclusion:**

Considering the increasing possibilities to move inside the European Union, is necessary to improve the European surgical formative system to warrant an uniform formation for all surgeons.

## Commentary

In the January issue of your journal there was an editorial [1] denouncing the grave problem regarding many surgeons' insufficient preparation when faced with emergency surgeries. Emergency surgery has become a neglected specialization in Europe and in many other parts of the world. In certain medical fields, emergency surgery isn't even considered an autonomous specialization. The flawed logic behind this idea is that every surgeon, skilled and proficient in his or her specific field of expertise, should also be capable of operating normally in the high stress environment of emergency surgery. However, this assertion is incontrovertibly false; this problem must be addressed, beginning with the restructuring of training programs for young surgeons. Both general surgery training and emergency surgery specialization must be crafted to better prepare surgeons for emergency interventions. Furthermore, every emergency surgeon should have substantial experience in general surgery before specializing.

The stark disparities between different European surgical formative systems are becoming increasingly distinct and recognizable. There are 27 individual countries in the European Union (EU), each of which having a distinctly different formative program. Some of these systems provide young surgeons with satisfactory theoretical and practical instructional backgrounds for the emergency surgery field. However, other less fortunate formative systems lack the support and training opportunities necessary to foster competent surgeons. If research were to be conducted, the results would inevitably demonstrate that the most stagnant and inflexible systems exist where there is the least amount of opportunities to learn and practice as a developing surgeon. This is common sense and hardly newsworthy, but it has dramatic implications for those dedicated and capable individuals who wish to improve their surgical skills, yet are hindered by such dysfunctional preparatory systems. The main problem is that certain systems do not mandate a minimum theoretical and practical understanding of a given field, whether initially during general surgery exercises or later during specialization. This instructional laxity is absolutely unacceptable and presents a notable hazard for the EU, considering that surgical certifications are reciprocally recognized between programs within all EU states. Every high-risk endeavour requires uniform preparation and training for its respective operatives, just as it is for the standardized emergency protocols regarding airports and airplanes. In this way, standardized courses of action are indoctrinated, thereby encouraging sensible responses when stressful environments prevent one from making calm, calculated decisions on an individual basis. Everyone would benefit from a unified system throughout the EU, one that has been scrupulously cross-examined by different parties to ensure high treatment standards. This could only be achieved by actively preparing medical students, the future doctors of tomorrow, for such a significant institutional transition.

One of the main problems of the aforementioned "lax system" is the absolute, incontestable authority conferred to its directors, a jurisdiction that can never be effectively challenged or disputed by surgeons in training. Furthermore, surgical students cannot choose between programs. Young impressionable surgeons are often forced to remain in the same facility for the duration of the formative program without having the opportunity to experience different systems and techniques, even if the instruction they receive is clearly inadequate. There is no independent oversight governing these programs and consequently no one is ever truly held accountable. Often, the very instructors themselves are the only individuals that scrutinize performance reviews, consider suggestions, or investigate complaints.

The EU as an institution has already experienced great political and economic success by embracing the poorer European states alongside their wealthier counterparts, thereby spreading prosperity across the continent. But what about cultural, formative, and scientific discrepancies? Shouldn't these be acknowledged as well? Why is it so difficult to continue taking innovative steps in this direction to pioneer a uniform system for medical students? There are many people ready and willing to make this transition; they need only the support of academic institutions and the ability to demonstrate the transition's effectiveness in an international context throughout the EU. Why don't we rally for a uniform European formative program to standardize the different systems, choosing the best qualities from each of them? Why don't we support an efficient and user-friendly exchange program for young surgeons who desire to broaden their professional and cultural horizons? Why don't we allow individuals to freely choose certain features of one's program, thereby creating a personalized curriculum that more closely reflects the needs and interests of a given student? Why don't we mandate that every young surgeon change his or her hospital at least once during their course of study to widen their professional perspectives? Perhaps these aren't the only solutions, but maybe they could begin to reinvigorate these stagnant systems, better preparing young surgeons both during general surgery training and later during specialization.

## Competing interests

As a Resident Surgeon and as a Student both willing to learn as much as possible to improve our theoretical and surgical skills, we tried to give our contribution to the improvement of a perfectible formative system. The authors declare that they have no financial competing interests

## Authors' contributions

Both authors gave substantive intellectual contributions to the elaboration of the article. F.C. resumed and elaborated the information from the different European formative systems. D.L. played an essential role on the evaluation of the information and on the definitive draft of the article. All authors read and approved the final manuscript.
